# Overweight and obesity epidemic in Ghana—a systematic review and meta-analysis

**DOI:** 10.1186/s12889-016-3901-4

**Published:** 2016-12-09

**Authors:** Richard Ofori-Asenso, Akosua Adom Agyeman, Amos Laar, Daniel Boateng

**Affiliations:** 1 Research Unit, Health Policy Consult, P. O. Box WJ 537, Weija, Greater-Accra Ghana; 2 Department of Population, Family and Reproductive Health, School of Public Health, University of Ghana, Legon, Accra, Ghana; 3 School of Public Health, Kwame Nkrumah University of Science and Technology, Kumasi, Ghana; 4 Julius Global Health, Julius Center for Health Sciences and Primary Care, University Medical Centre, Utrecht University, Utrecht, The Netherlands

**Keywords:** Obesity, Nutrition transition, Non-communicable diseases, Ghana, Meta-analysis

## Abstract

**Background:**

In many low and middle income countries (LMICs), the distribution of adulthood nutritional imbalance is shifting from a predominance of undernutrition to overnutrition. This complex problem poses a huge challenge to governments, non-state actors, and individuals desirous of addressing the problem of malnutrition in LMICs. The objective of this study was to systematically review the literature towards providing an estimate of the prevalence of overweight and obesity among adult Ghanaians.

**Methods:**

This study followed the recommendations outlined in the PRISMA statement. Searches were performed in PubMed, Science Direct, google scholar, Africa Journals Online (AJOL) and the WHO African Index Medicus database. This retrieved studies (published up to 31st March 2016) that reported overweight and obesity prevalence among Ghanaians. All online searches were supplemented by reference screening of retrieved papers to identify additional studies.

**Results:**

Forty-three (43) studies involving a total population of 48,966 sampled across all the ten (10) regions of Ghana were selected for the review. Our analysis indicates that nearly 43% of Ghanaian adults are either overweight or obese. The national prevalence of overweight and obesity were estimated as 25.4% (95% CI 22.2–28.7%) and 17.1% (95% CI = 14.7–19.5%), respectively. Higher prevalence of overweight (27.2% vs 16.7%) and obesity (20.6% vs 8.0%) were estimated for urban than rural dwellers. Prevalence of overweight (27.8% vs 21.8%) and obesity (21.9% vs 6.0%) were also significantly higher in women than men. About 45.6% of adult diabetes patients in Ghana are either overweight or obese. At the regional level, about 43.4%, 36.9%, 32.4% and 55.2% of residents in Ashanti, Central, Northern and Greater Accra region, respectively are overweight or obese. These patterns generally mimic the levels of urbanization. Per studies’ publication years, consistent increases in overweight and obesity prevalence were observed in Ghana in the period 1998–2016.

**Conclusions:**

There is a high and rising prevalence of overweight and obesity among Ghanaian adults. The possible implications on current and future population health, burden of chronic diseases, health care spending and broader economy could be enormous for a country still battling many infectious and parasitic diseases. Public health preventive measures that are appropriate for the Ghanaian context, culturally sensitive, cost-effective and sustainable are urgently needed to tackle this epidemic.

**Electronic supplementary material:**

The online version of this article (doi:10.1186/s12889-016-3901-4) contains supplementary material, which is available to authorized users.

## Background

For decades, undernutrition has been the focus of nutrition agendas in many countries, particularly in low and middle income countries (LMICs). Whereas infectious and parasitic diseases remain major unresolved health problems in many LMICs, emerging non-communicable diseases (NCDs) relating to diet, lifestyle, and overweight/obesity have been increasing over the last three decades [1]. The influence of the demographic transition, epidemiologic transition, and currently nutrition transition on the current state of global health is well characterized [2].

The nutrition transition is characterized by a shift in disease burden from undernutrition to overnutrition-related chronic diseases. The key drivers of nutrition transition include economic development and rapid urbanization that facilitates shifts in dietary patterns from traditional diets such as those rich in complex carbohydrates and fiber to energy-rich foods high in fat and sweeteners [3, 4]. This alongside increased sedentary lifestyle lead to obesity and related chronic diseases [5, 6]. In many developing countries, the rising over-nutrition comes along with significant burden of under-nutrition, and multiple micronutrient deficiencies resulting in a complex “multiple burden of malnutrition” [7].

According to the World Health Organization (WHO), obesity remains “one of today’s most blatantly visible–yet most neglected–public health problems” [8]. Prevalence of obesity across the world has increased more than 200% since 1980 with nearly 2 billion adults estimated to be overweight in 2014 including 600 million individuals who were obese [9]. On the other hand, the prevalence of under-nutrition has not changed significantly over the last decade [10].

Overweight and obesity are used to represent abnormal or excessive fat accumulation that has the potential to exert negative effects on health [8, 11]. Obesity occurs when calories intake exceeds the energy requirements of the body both for physical activity and for growth [12]. The increasing prevalence of obesity is widely attributed to genetic factors, changes in dietary and physical activity patterns and the increasing availability of high fatty foods [11, 12]. As noted earlier, the driving forces behind these trends include globalization, which is recognized to be dictating a widespread “nutrition transition” in many countries characterized by a shift from traditional to western diets and an increasing sedentary lifestyle [13, 14].

The nutritional status of a person is frequently described by the Body Mass Index (BMI), which is the ratio of the weight (Kg) and the square of height (metres). Over the years, the BMI has widely been used to assess overweight and obesity in adults [9]. Individuals are categorized according to BMI as follows; underweight (BMI <18.5 kg/m^2^); normal (18.5–24.9 kg/m^2^); overweight (25.0–29.9 kg/m^2^) and obese (≥30 kg/m^2^) [15, 16].

Underweight, overweight and obesity are known risk factors for NCDs [17, 18]. Raised BMI (overweight/obesity) is a major risk factor for cardiovascular diseases, diabetes, hypertension, musculoskeletal disorders and some cancers [11–13]. The overall impact of obesity/overweight on physical and mental health as well as health-related quality of life is significant [19]. In countries where the economic impact of obesity has been studied, its direct (arising from preventive, diagnostic, and treatment services) and indirect (related to decreased productivity, increased absenteeism and restricted activity etc.) costs have been estimated to be enormous. In the UK, the direct and indirect costs of obesity have been estimated to be far in excess of 2 billion pounds sterling per year [20], while in the US, absenteeism arising from obesity alone has been estimated to cost as much as $4.3 billion annually [21].

Once conditions of the developed world, overweight and obesity are now prevalent in many low- and middle-income countries [22, 23]. In Sub-Saharan Africa, the rising prevalence of overweight and obesity co-exists with the under-nutrition epidemic [24, 25] and the increasing prevalence of NCDs with an anticipated largest increase in NCD deaths of 27% in Africa over the next decade [26].

In Ghana, obesity/overweight have been recognized to be increasing public health problem that could impact significantly on national resources [27, 28]. The Ghana Demographic and Health Surveys (GDHS) from 1993 to 2014 reported an increasing prevalence of obesity among Ghanaian women (15–49 years) from 3.4% to 15.3% [29–31]. The WHO estimates that in 2008, around 7.5% of Ghanaians were obese with higher prevalence in women (10.9%) than men (4.1%) [32]. A meta-analysis including studies from Ghana reported an obesity prevalence of 10% among adults in West Africa, with women being three times more likely to be obese than men (odds ratio = 3.16). However, this may not fully represent the pertaining Ghanaian situation as almost half of all the included studies (46.4%) in the review were from Nigeria [10]. In our literature search, we identified one systematic review by Commodore-Mensah et al. [33], which reported overweight/obesity prevalence in Ghanaian adults as within the range 20–62%. Aside the wide range presented, this review involved only nine (9) studies published no later than June 2012 and excluded high risk groups as well as studies conducted in hospital settings. While the 2013 global burden of disease study also reported overweight and obesity prevalence in Ghanaian adult (>20 years) males (overweight = 15 · 4%; obesity = 2.5%) and females (overweight = 29.1%, obesity = 9.8%), these estimates were based on data selected from only nine survey reports [26]. Additionally, none of the previously published reviews provide clues as to the temporal changes in overweight and obesity prevalence in Ghana.

Our general observation points to a lack of thorough systematic review of the literature towards documenting the prevalence of overweight and obesity in Ghana. To support evidence-based policymaking, resource allocation and the design of appropriate public health interventions, accurate overweight and obesity prevalence estimates based on thorough and up-to-date evidence compilation is urgently needed. In this study, we conducted a systematic review to summarize the available information to date towards estimating the prevalence of overweight and obesity among Ghanaian adults. Additionally, we sought to assess the temporal changes in overweight and obesity prevalence in the country.

## Methods

This review adhered to the recommendations outlined in the PRISMA (Preferred Reporting Items for Systematic Reviews and Meta-Analyses) statement [34] (Additional file 1).

### Search strategy

Searches were conducted in PubMed, Science Direct, google scholar, Africa Journals Online (AJOL) and the WHO African Index Medicus database to retrieve primary studies reporting overweight and obesity prevalence among Ghanaians. The keywords used in our searches were “obesity OR overweight OR anthropometry OR adiposity” AND “prevalence” AND “Ghana OR Ghanaian”. All searches were conducted between 20/03/2016 and 31/03/2016 by RO and AAA. References of all selected papers were screened along with those of previously published reviews with the aim of identifying additional studies which may have been missed through our online searches.

### Inclusion and exclusion of studies

We included studies published up to 31st March 2016 which reported overweight and obesity prevalence among Ghanaians adults (≥18 years). RO and AAA conducted titles and abstract screening against the pre-defined study inclusion criteria. Additionally, full-text articles were also independently screened by the same reviewers (AAA, RO) for eligibility. To allow for aggregation/pooling of data, we only included studies that used BMI to define overweight and obesity prevalence. We classified BMI of 25–29.9 kg/m^2^ and ≥ 30 kg/m^2^ to represent overweight and obesity in adults, respectively [15, 16]. Studies were included only if BMI was stratified and separate prevalence of overweight and/or obesity were presented. We excluded studies conducted in children as the focus in this review was on adults. Studies presenting self-reported overweight and obesity prevalence were excluded as these have been found to usually underestimate overweight and obesity prevalence [35, 36]. For studies with multiple publications, the version published first or one with complete dataset was selected. We assessed the quality of studies, based on a 12-point scoring system adopted from the Downs and Black checklist [37]. For each study, descriptive details such as author details, publication year, region where study was conducted, study design, type of setting (e.g. rural), the study population, mean age of participants and the overweight and obesity prevalence rates were collected. Data were independently extracted by RO, AAA and DB and crosschecked. Any disparities in data were resolved by consensus-based discussions among the authors.

### Data analysis

Statistical analysis and meta-analysis proportions were carried out using OpenMeta (analyst) software, an open-source, cross-platform software for advanced meta-analysis [38] and StatsDirect statistical software (Version 3.0.0, StatsDirect Ltd, Cheshire UK) [39]. The pooled effects and individual study proportions were assessed at 95% confidence interval (CI). We performed heterogeneity test for all the proportions based on Cohran’s heterogeneity statistic (Q) and degree of inconsistency (I^2^) [40]. I^2^ > 50% was considered to denote meaningful heterogeneity in which case the random effect model (DerSimonian-Laird) was used instead of the fixed effect model in the analysis of pooled effects [40]. The presence of publication bias was assessed by direct observation of funnel plots as well as through Egger and Begg’s regression tests [41, 42]. We assessed the robustness of the pooled estimates by conducting a leave-one-out sensitivity analysis to assess the impacts that each study exerts on the overall pooled estimate [43]. Furthermore, sub-analysis was carried out across gender and settings (urban vs rural), across studies’ publication periods as well as for specific populations such as persons with diabetes. In all analysis, we considered statistical significance to represent *p* < 0.05.

## Results

### Overview of studies

**Fig. 1:**
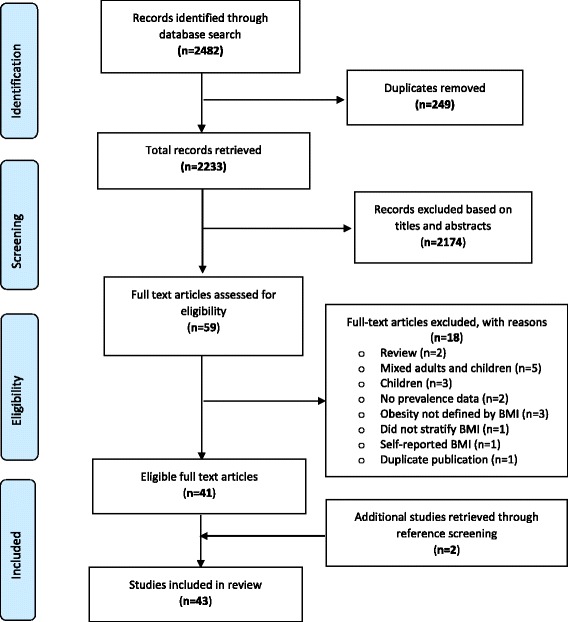
PRISMA flow chart of the studies’ identification steps

Figure 1 represents the PRISMA flow chart outlining the steps in retrieving appropriate studies for the review. A total of 2482 citations were identified through electronic search and other sources. After the exclusion of duplicates and assessment of titles and abstracts, 59 articles were shortlisted for detailed full-text analysis. Out of this number, 41 met the inclusion criteria for addition to the review. Two (2) additional studies were retrieved through reference screening of selected papers bringing the total number of studies included in the review to 43 [44–86]. These 43 studies (Table 1) involved a sample population of 48,966 with individual study sample size ranging from 59 to 9215. The studies were conducted across all the 10 regions of Ghana and included 4 nationally-sampled studies [60, 70, 76, 86]. Regional-based studies were distributed as follows; Ashanti (n = 11), Greater Accra (n = 15), Northern (n = 7), Volta (n = 1), Upper East (n = 1), Central (n = 3) and one inter-regional study (Greater Accra and Upper West). While twelve (12) studies did not report the actual period (year/s) in which sampling was conducted, in the remaining 31 studies sampling were conducted between 1988 and 2016. Per publication years, 93% (n = 40) of studies were published within the last decade (2006–2016) and 79% (n = 34) were published within the last 5 years (2011–2016). Applying the quality assessment criteria, 56%, 35% and 9% of studies were graded as high, medium and low quality, respectively.

**Table 1:** Summary of the descriptive characteristics of individual studies

Study No.	Author	Year of Publication	Sampling Period	Region	Study design	Setting	Study population	Mean age of participants (years)	Sample size (n)					Quality grade
									All	M	F	R	U	
1.	Abubakari et al. [44]	2015	2014	Northern	Cross-sectional (facility-based)	Mixed	Pregnant Women + new mothers	n.s	419	-	419	-	-	Medium
2.	Addo et al. [45]	2006	n.s	Greater Accra	Cross-sectional	Rural	General adult Population	42.4	362	107	255	362	-	High
3.	Addo et al. [46]	2009	2006	Greater Accra	Cross-sectional	Urban	Civil servants	n.s	1015	615	400	-	1015	High
4.	Addo et al. [47]	2015	n.s	Greater Accra	Cross-sectional	Urban	Bank workers	32.2	180	92	88	-	180	Medium
5.	Agyemang [48]	2006	2004	Ashanti	Cross-sectional	Rural and urban	General Population	35.9	1431	644	787	578	853	High
6.	Aidoo et al. [49]	2015	2012	Greater Accra	Cross-sectional	Urban	Corporate workers	45.1	161	112	49	-	161	Low
7.	Amegah et al. [50]	2011	n.s	Central	Cross-sectional	Urban	General population	n.s	300	150	150	-	300	Medium
8.	Amidu et al. [51]	2016	2014	Northern	Cross-sectional	Urban	Christian worshippers	34.53	300	133	167	-	300	medium
9.	Amoah [52]	2003	n.s	Greater Accra	Cross-sectional	Rural and Urban	General adult population (≥25 yrs)	44.3	4731	1857	2874	1627	3104	High
10.	Antwi et al. [53]	2016	2012	Greater Accra and Upper west	Retrospective record review	Rural and Urban	Pregnant women	27.0	2682^a^	-	2682	1104	1578	Medium
11.	Appiah et al. [54]	2014	n.s	Ashanti	Cross sectional	Urban	Women	43.8	394	-	394	-	394	Medium
12.	Arthur et al. [55]	2013	2011	Ashanti	Cross-sectional	Urban	Menopausal and pre-menopausal women	44.23	250	-	250	-	250	Medium
13.	Arthur et al. [56]	2014	2013	Ashanti	Cross-sectional	Urban	University staff	n.s	206	138	68	-	206	High
14.	Aryee et al. [57]	2013	n.s	Northern	Cross-sectional	Urban	Nurses	n.s	220	74	146	-	220	Medium
15.	Aryeetey and Ansong [58]	2011	2009	Greater Accra	Cross-sectional	Urban	University staff	40.5	141	95^a^	46^a^	-	141	Low
16.	Benkeser et al. [59]	2012	2008-09	Greater Accra	Cross-sectional	Urban	women	46.28	2814	-	2814	-	2814	High
17.	Biritwum et al. [60]	2005	2003	National	Cross-sectional	Rural and Urban	General population	-	4231	-	-	-	-	High
18.	Blankson and Hall [61]	2012	2010	Ashanti	Cross-sectional	Rural	Elderly women	n.s	59	-	59	59	-	Low
19.	Burket [62]	2006	2002	Volta	Cross-sectional	rural	General population	41.8	284	66	218	284	-	High
20.	Donkor et al. [63]	2015	n.s	Greater Accra	Cross sectional	Urban	Women	n.s	254	-	254	-	254	Medium
21.	Duda et al. [64]	2006	2005	Greater Accra	Cross-sectional (hospital based)	Urban	Women at gynecology clinic	35.9	305	-	305	-	305	High
22.	Duda et al. [65]	2007	2003	Greater Accra	Cross-sectional	Urban	Women	46.8	1270	-	1270	-	1270	High
23.	Ephraim et al. [66]	2014	2013-14	Central	Case-control	Urban	Pregnant women	30.9	380	-	380	-	380	Medium
24.	Frank et al. [67]	2013	2007-08	Ashanti	Cross-sectional (facility-based)	Urban	Diabetic and Non-diabetic adults	n.s	1221	299	922	-	1221	medium
25.	Kunutsor and Powles [68]	2014	n.s	Upper East	Cross-sectional	Rural	General population	37.8	574	207	367	574	-	Medium
26.	Luke et al. [69]	2014	2010-11	Ashanti	Cross-sectional	Rural	Young adults	34.6	500	207	293	500	-	High
27.	Minicuci et al. [70]	2014	2007-08	National	Cross-sectional	Rural Urban	Older adults (≥50)	n.s	4724	2348	2376	2806	1918	High
28.	Mogre et al. [71]	2014	2013	Northern	Cross-sectional	Urban	University students	23.06	646	445	201	-	646	High
29.	Mogre et al. [72]	2014	2013	Northern	Cross-sectional (facility-based)	Urban	Diabetics	56.2	200	46	154	-	200	High
30.	Mogre et al. [73]	2015	n.s	Northern	Cross-sectional	Urban	University students	23.0	552	354	198	-	552	High
31.	Mogre et al. [74]	2016	2014	Northern	Cross-sectional (facility-based)	Urban	Diabetics	47.3	378	132	246	-	378	High
32.	Nelson et al. [75]	2015	2010	Greater Accra	Cross-sectional (facility-based)	Urban	Hospital Outpatients	n.s	230	125	105	-	230	Medium
33.	Nubé et al. [76]	1998	1988-89	National	Cross-sectional	Mixed	General Population (20–65)	n.s	9215	4961	4253	5788	3427	High
34.	Nyawornota et al. [77]	2013	n.s	Greater Accra	Cross-sectional	Urban	Students	18.4^a^	155^a^	-	-	-	155^a^	Low
35.	Obirikorang et al. [78]	2015	2013	Ashanti	Cross-sectional	Rural and Urban	General population	50.0	672	312	360	360	312	High
36.	Obirikorang et al. [79]	2016	2014-15	Ashanti	Cross-sectional (facility-based)	Urban	Diabetics	51.14	543	232	311	-	543	Medium
37.	Oppong et al. [80]	2015	2013	Greater Accra	Cross-sectional (facility-based)	Urban	Pregnant women	31.0	399	-	399	-	399	High
38.	Owiredu et al. [81]	2011	2010	Ashanti	Case-control	Urban	Sportsmen and sedentary workers	43.56	186	-	-	-	186	Medium
39.	Pereko et al. [82]	2013	n.s	Central	Cross-sectional	Urban	Adults	31.7	252	101	151	-	252	High
40.	Pobee et al. [83]	2013	2006	Greater Accra	Cross-sectional	Urban	Female teachers	35.9	400	-	400	-	400	High
41.	Van der Linden et al. [84]	2016	2012-14	Greater Accra	Cohort study	Urban	Pregnant women	28.0	1000	-	1000	-	1000	High
42.	Williams et al. [85]	2013	n.s	Ashanti	Cross-sectional	Rural	Adults ≥35	50	425	152	273	425	-	Medium
43.	Wu et al. [86]	2015	2007-10	National	Longitudinal cohort survey	Rural and Urban	Ault population ≥50	n.s	4305	2256	2049	2536	1769	High

*M* male, *F* female, *R* rural, *U* urban, ^a^ calculated from study datam, *n.s*. not specified

### Overweight prevalence among Ghanaian adults

**Fig. 2:**
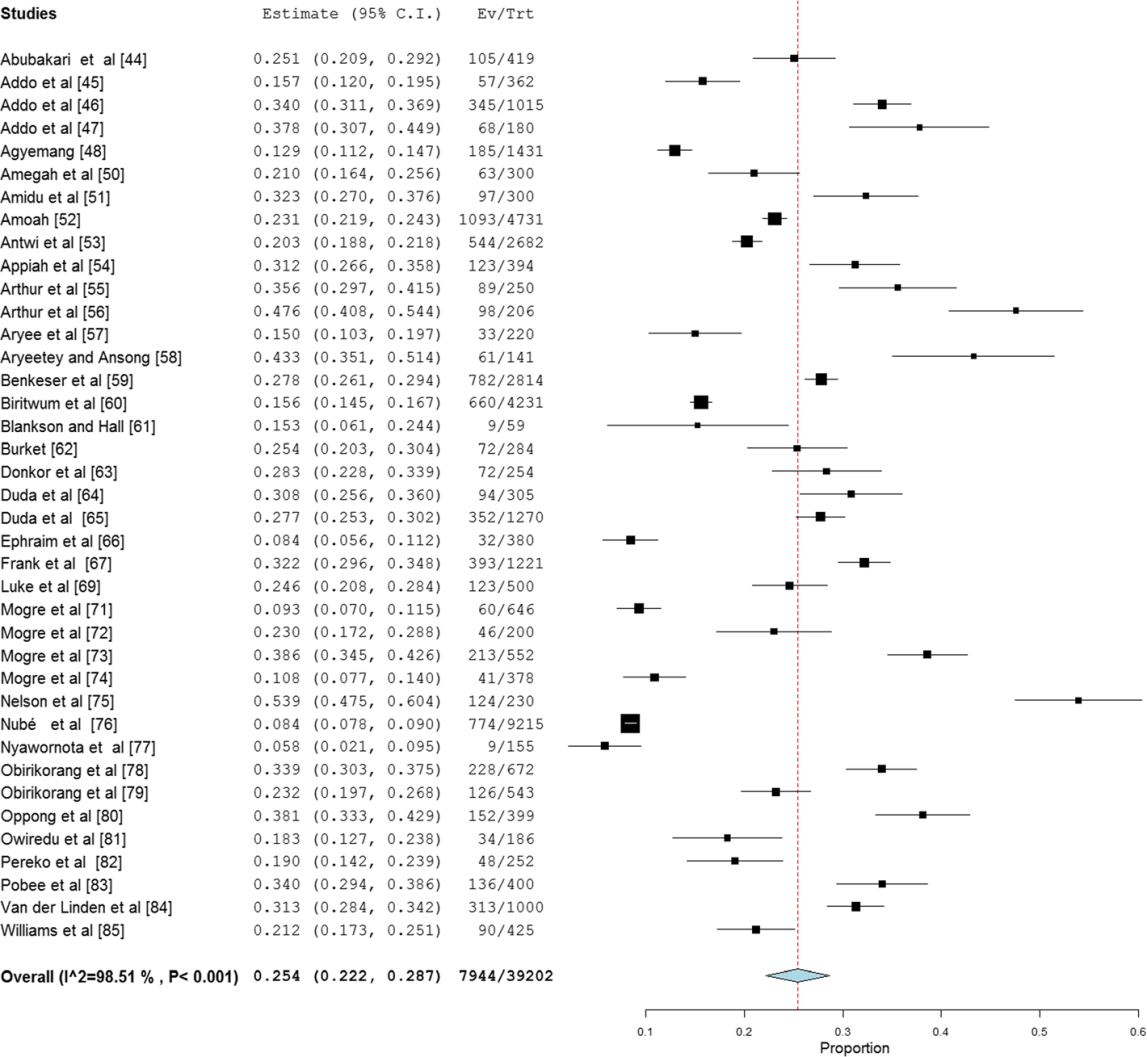
Forest plot of studies reporting overweight prevalence among Ghanaian adults

**Fig. 3:**
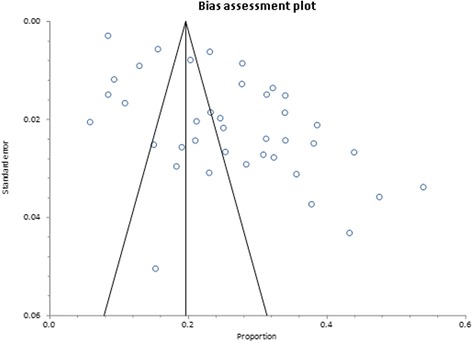
Funnel plot of studies reporting overweight prevalence among Ghanaian adults

**Fig. 4:**
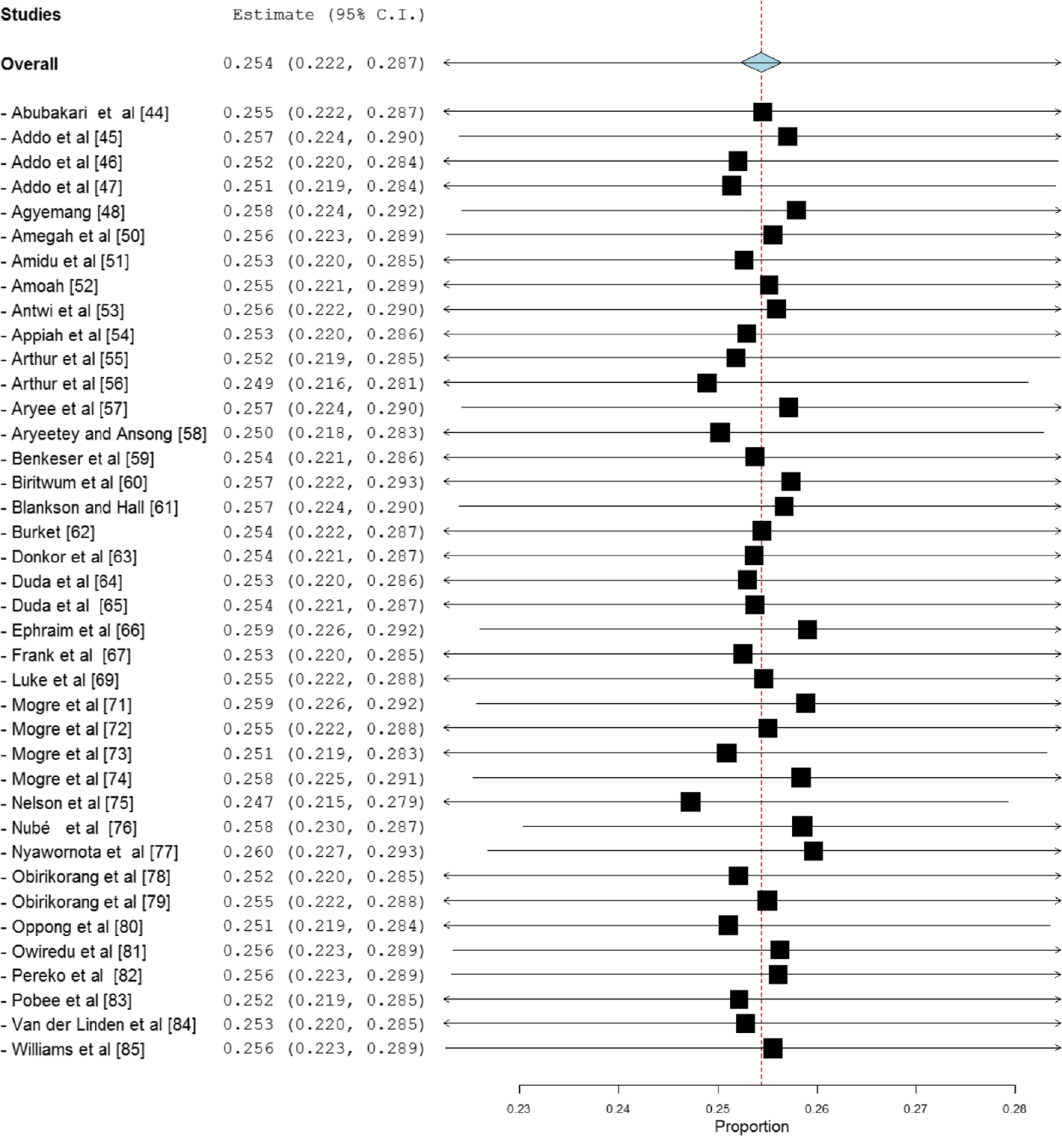
A leave-one-out sensitivity plot of studies reporting overweight prevalence among Ghanaian adults

Overweight prevalence data was retrieved from 39 studies with a combined sample size of 39,202. Among these studies, the reported overweight prevalence ranged from 5.8% to 54.0%. The pooled national prevalence of overweight (Fig. 2) among Ghanaian adults from the 39 studies was estimated as 25.4% (95% CI 22.2–28.7%). I^2^ was determined as 98.51% (*p* < 0.001) for the degree of inconsistency. A funnel plot of the overweight prevalence showed presence of publication bias as depicted by an asymmetrical display of prevalence reported by various studies (Fig. 3). This was confirmed by an Egger’s test which was significant (*p* < 0.0001). A leave-one-out sensitivity analysis also revealed that the pooled estimate was most impacted by prevalence data from Nelson et al. [75] (Fig. 4).

Overweight prevalence among males was determined as 21.8% (95% CI = 16.2–27.4%; I^2^ = 98.43%, *p* < 0.001). Among females, overweight prevalence information was collected from thirty (30) studies with a total sample size of 22,079. Reported overweight prevalence for females from these studies was within the range 8.4–66.6% and the pooled estimate was determined as 27.8% (95% CI 24.4–31.3%; I^2^ = 97.27%, *p* < 0.001). The difference (6.0%; 95% CI 5.0–7.0%) in overweight prevalence between males and females was statistically significant (*p* < 0.0001).

Overweight prevalence among adults with diabetes was estimated using data from 4 studies with a combined sample size of 1663. The pooled overweight prevalence among the diabetics was estimated as 30.0% (95% CI = 22.1–37.8%; I^2^ = 91.89%, *p* < 0.001).

Overweight prevalence among rural dwellers was estimated using data from nine (9) studies with a combined sample size of 10,803. The overweight prevalence was within the range 4.8–43.9%. The pooled overweight prevalence in rural dwellers was estimated as 16.7% (95% CI 11.2–22.3%; I^2^ = 98.3%, *p* < 0.001). Overweight prevalence among urban dwellers was estimated using information from 32 studies that together involved a total sample population of 23,465. Among the 32 studies the overweight prevalence among urban dwellers was within the range 5.8–54%. The pooled overweight prevalence among urban dwellers was estimated as 27.2% (95% CI 23.7–30.7%; I^2^ = 97.53%, *p* < 0.001). The difference (10.5%, 95% CI 9.6–11.4%) in overweight prevalence between urban and rural dwellers was statistically significant (*p* < 0.0001).

Overweight prevalence for Ashanti, Greater Accra, Northern and Central regions were estimated based on data from 11 (sample population size = 5887), 15 (sample population size = 14,834), 7 (sample population size = 2715) and 3 (sample population size = 932) studies respectively, as follows; Ashanti = 26.9% (95% CI 20.7–33.0; I^2^ = 96.65%, *p* < 0.001); Greater Accra = 30.1% (95% CI 26.2–33.9; I^2^ = 96.1%, *p* < 0.001); Northern = 21.9% (95% CI 13.8–29.9; I^2^ = 96.87% *p* < 0.001) and Central region = 16.0% (95% CI 7.3–24.7%; I^2^ = 92.82%, *p* < 0.001). We were unable to estimate overweight prevalence for the six (6) remaining regions due to limited number of studies reporting prevalence for these regions.

### Obesity prevalence among Ghanaian adults

**Fig. 5:**
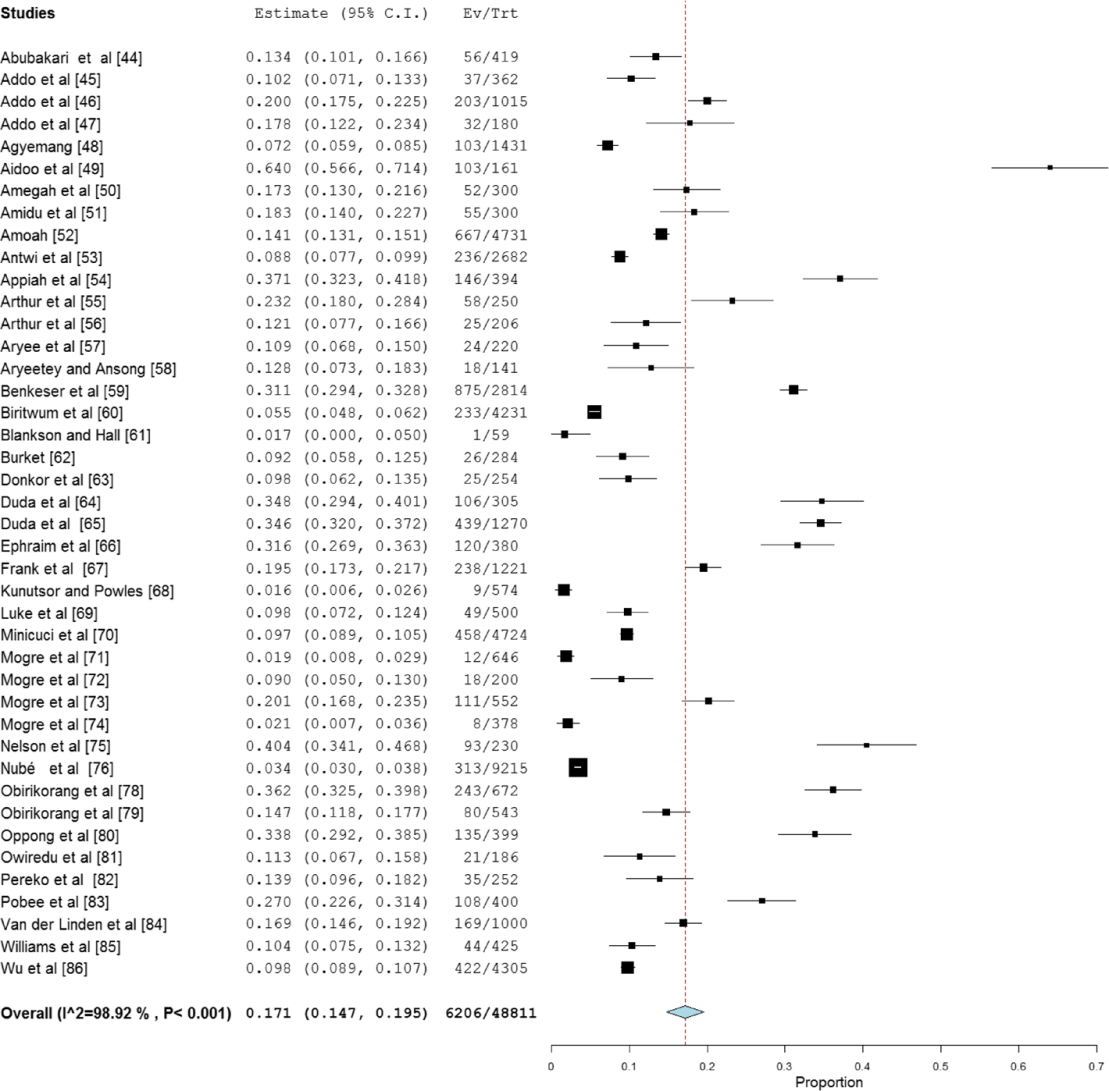
Forest plot of studies reporting obesity prevalence among Ghanaian adults

**Fig. 6:**
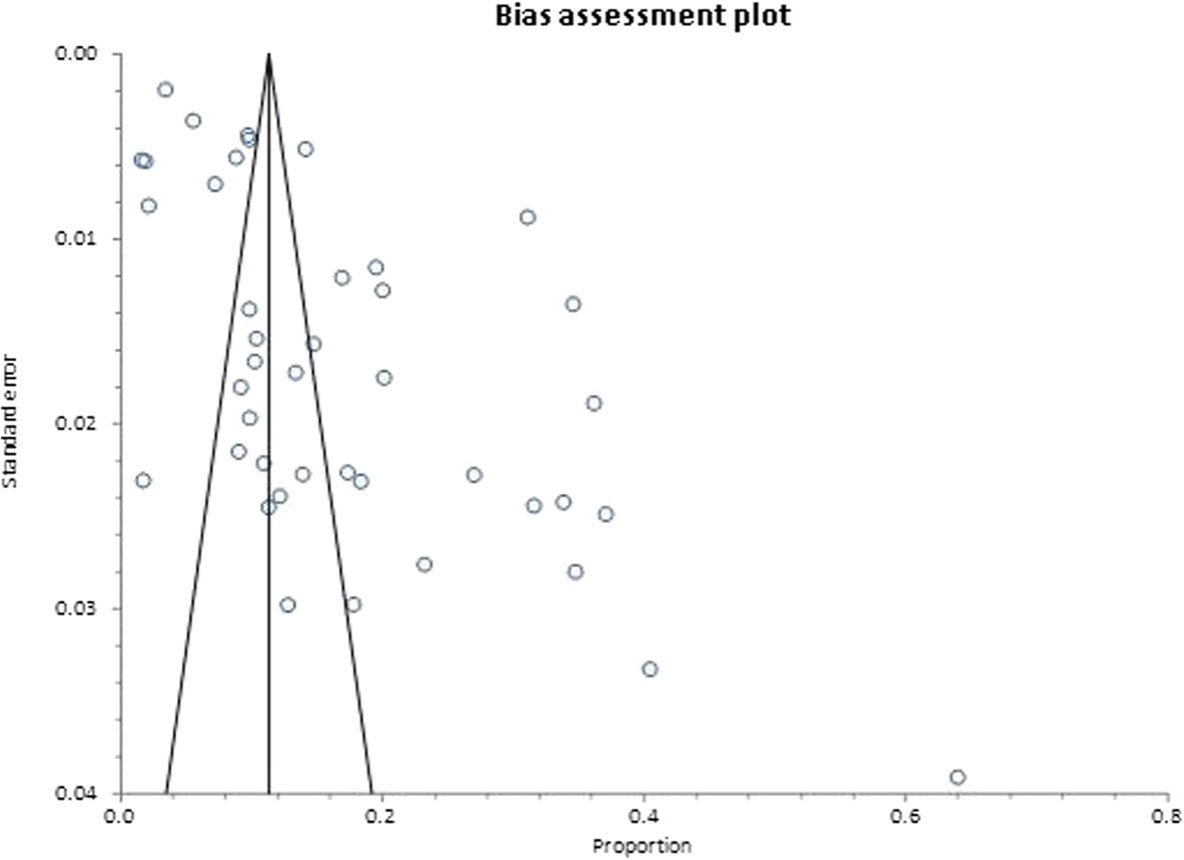
Funnel plot of studies reporting obesity prevalence among Ghanaian adults

**Fig. 7:**
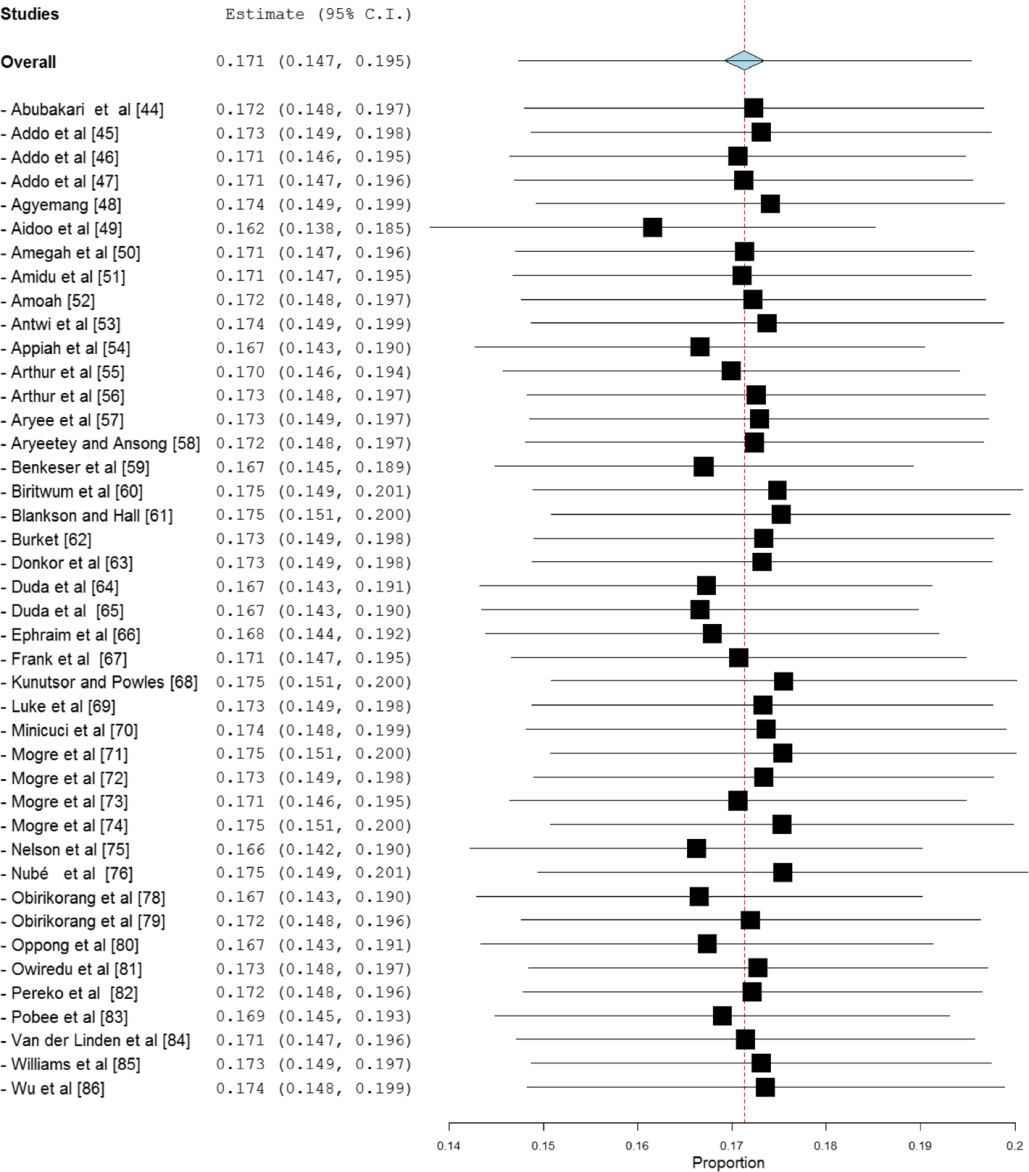
A leave-one-out sensitivity plot of studies reporting obesity prevalence among Ghanaian adults

Obesity prevalence data was retrieved from forty-two (42) studies with a combined sample size of 48,811. Among these studies, the reported obesity prevalence was within the range 1.6–63.8%. The pooled national prevalence of obesity (Fig. 5) among Ghanaian adults based on the 42 studies was estimated as 17.1% (95% CI = 14.7–19.5%). I^2^ was determined as 98.9% (*p* < 0.001) for the degree of inconsistency. A funnel plot of obesity prevalence rates showed presence of publication bias as depicted by an asymmetrical display of prevalence reported by various studies (Fig. 6). This was confirmed by an Egger’s test which was significant (*p* < 0.0001). A leave-one-out analysis revealed that the pooled obesity prevalence estimate was most impacted by Aidoo et al. [49] (Fig. 7).

Obesity prevalence among males was retrieved from seventeen (17) studies with a total sample population size of 10,550. These 17 studies reported obesity prevalence in the range 0.6–38%. The pooled obesity prevalence estimate for males was estimated as 6.0% (95% CI 4.4–7.6%; I^2^ = 95.3%; *p* < 0.001). Obesity prevalence among females was retrieved from twenty-nine (29) studies with a total sample size of 21,079. These 29 studies reported obesity prevalence in the range 2–55%. The pooled obesity prevalence estimate was determined as 21.9% (95% CI 17.7–26.2%; I^2^ = 98.74%, *p* < 0.001). The difference (15.9%; 95% CI 15.2–16.6%) in obesity prevalence between males and females was statistically significant (*p* < 0.0001).

Among diabetes patients, the obesity prevalence was estimated using data from 4 studies with a combined sample size of 1663. The pooled obesity prevalence estimate was determined 15.6% (95% CI 11.2–20.0%; I^2^ = 83.92%, *p* < 0.001).

Prevalence of obesity in rural settings was estimated using data from 11 studies with a total sample population of 13,913 and individual prevalence rates ranging from 1.4% to 40.5%. The pooled obesity prevalence estimate for rural dwellers was estimated as 8.0% (95% CI 5.4–10.5%; I^2^ = 97.9%; *p* < 0.001). For urban dwellers, obesity prevalence data was retrieved from 33 studies with a total sample population of 25,240. The individual prevalence reported was within the range 1.9–63.8% and the pooled estimate was determined as 20.6% (95% CI 16.9–24.3%; I^2^ = 98.99%, *p* < 0.001. The difference (12.6%, 95% CI 11.9–13.2%) in obesity prevalence between urban and rural dwellers was found to be statistically significant (*p* < 0.0001).

**Fig. 8:**
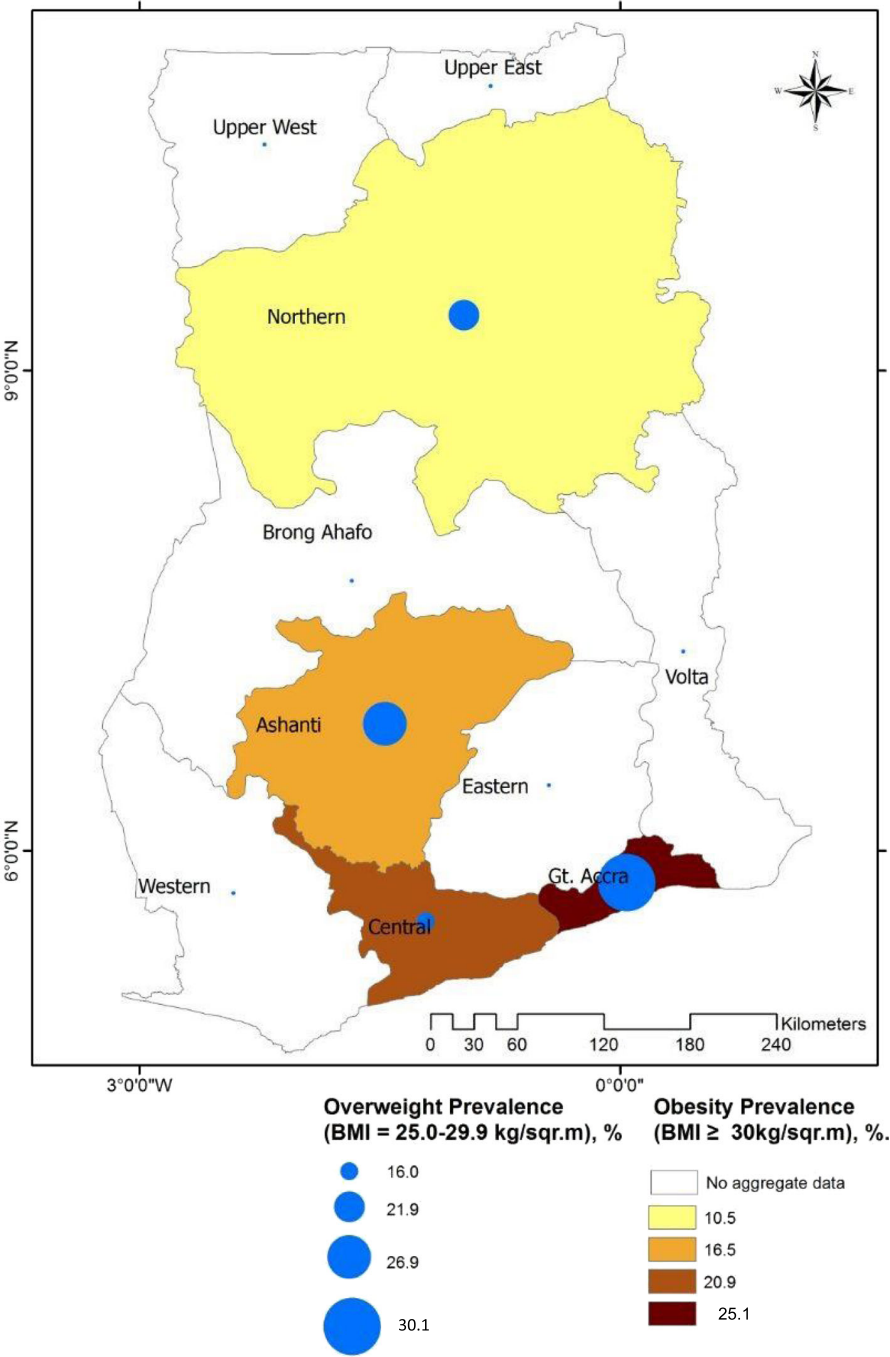
Regional prevalence of overweight and obesity among adults in Ghana

Obesity prevalence for Ashanti, Greater Accra, Northern and Central regions were estimated based on data from 11 (sample population size = 5887), 16 (sample population size = 14,840), 7 (sample population size = 2715) and 3 (sample population size = 932) studies respectively, as follows; Ashanti 16.5% (95% CI 10.8–22.3%; I^2^ = 97.67%, p < 0.0001); Greater Accra 25.1% (95% CI 20.0–30.1; I^2^ = 98.2%, *p* < 0.0001); Northern region 10.5% (95% CI 5.9–15.2; I^2^ = 96.59% P < 0.0001) and Central region = 20.9% (95% CI 10.6–31.2; I^2^ = 93.92% *p* < 0.001). We were unable to deduce obesity prevalence estimates for the remaining seven regions of Ghana due to limited number of individual prevalence data from studies (Fig. 8).

### Temporal changes in overweight and obesity prevalence among Ghanaian adults

**Fig. 9:**
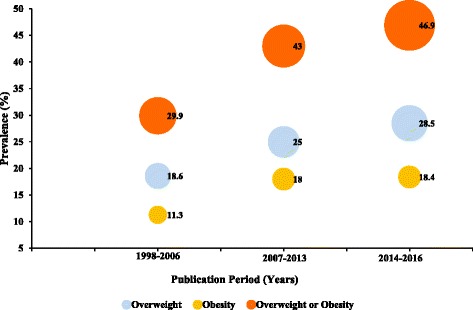
Overweight and obesity prevalence in Ghana according to studies’ publication years

As 28% (n = 12) of studies did not report the actual period (year/s) in which sampling were carried out, we resorted to a temporal analysis based on years in which studies were published. We grouped studies into the publication periods 1998–2006, 2007–2013 and 2014–2016 with a distribution of 7, 14 and 22 studies, respectively. The overweight prevalence for the periods 1998–2006, 2007–2013 and 2014–2016 were estimated as 18.6% (95% CI 13.1–24.1%; I^2^ = 99.0%, *p* < 0.0001), 25.0% (95% CI 20.6–29.3%; I^2^ = 95.04%, *p* < 0.0001) and 28.5% (95% CI 23.3–33.7%; I^2^ = 97.44%, *p* < 0.0001), respectively. Thus, the ascending order of overweight prevalence among Ghanaian adults according to studies’ publication period was 1998–2006 < 2007–2013 < 2014–2016. Obesity prevalence for the periods 1998–2006, 2007–2013 and 2014–2016 were also estimated as 11.3% (95% CI 7.6–15.0%; I^2^ = 98.9%, *p* < 0.0001), 18.0% (95% CI 12.4–23.6%; I^2^ = 97.57%, *p* < 0.0001), and 18.4% (95% CI 15.0–21.7%; I^2^ = 98.56%, *p* < 0.0001), respectively. Hence the ascending order of obesity prevalence among Ghanaian adults according to studies publication period was 1998–2006 < 2007–2013 < 2014–2016 (Fig. 9).

## Discussion

This review has documented a high prevalence of overweight (25.4%) and obesity (17.1%) among Ghanaian adults. Higher prevalence of overweight and obesity across studies published in the most recent years (2007–2016) as opposed to those published in earlier years (1998–2006) by inference highlight the growing burden of overweight and obesity in the country. The rising overweight and obesity burden as observed in this review is in line with observations already communicated by other researchers in the country [28, 87] and is further supported by the reported consistent increase in obesity/overweight prevalence by the Ghana DHS in the period 1988–2014 [29–31]. Our results and those of others all suggest and corroborate the fact that obesity is no more an issue of only “affluent nations” but becoming an increasing public health problem in LMICs such as Ghana.

For decades, undernutrition has traditionally been the focus of nutrition agendas in many LMICs. Whereas common infectious and parasitic diseases (e.g. Malaria, HIV and TB) remain major unresolved health problems in many LMICs [88], emerging NCDs relating to diet and lifestyle have been increasing over the last three decades; often times attributed to the demographic transition, epidemiologic transition, and currently nutrition transition [2, 89]. Facilitated by rapid economic development and urbanization [5], the nutrition transition is characterized by a shift in disease burden from undernutrition to overnutrition-related chronic diseases. Increased consumption of energy-dense foods and the lack of physical activity, which are marked characteristics of advancing nutrition transition, lead to obesity and the development of numerous chronic diseases. This has led to an increase in overweight and obesity and their-related chronic diseases [6].

While our analysis points to a growing problem of obesity, undernutrition still poses significant threat to health and wellbeing of many Ghanaians. In the 2014 DHS for instance, over 6% of Ghanaian women were found to be underweight [29]. This co-existence of undernourishment and overnutrition is a real public health challenge particularly among lower socioeconomic groups [90], who may lack the financial resources to avoid micronutrient-poor diet but may also be more likely to consume cheaper processed and energy-dense meals [91]. Evidence suggests that in many LMICs, increasing numbers of lower socioeconomic groups struggle with undernutrition, even as obesity and overnutrition increases [92].

Several theories (such as life-course perspective) have been proposed to explain this phenomenon of rising obesity among populations with systemic undernutrition challenges [89]. Amuna and Zotor [93], explain that fetal exposure to maternal malnutrition during pregnancy could increase risk of nutritional disorders in later life. Another explanation also points to the role that poor childhood nutrition (i.e. stunting, underweight) could impact future physiological pathways. For instance, poor nutritional status during early stages of development may boost the development of thrifty phenotype that may increase risks of chronic conditions including obesity, diabetes and cardiovascular diseases in later life [94–96]. Additionally, infections contracted at early life years can also increase future risk of NCDs such as obesity [89, 97]. This may be an important component particularly in LMICs were infectious diseases remain highly prevalent and children are often at high risk [98, 99].

In this review, we report a 1.3 times higher prevalence of overweight and about 3.7 times higher prevalence of obesity in women as compared to men. This trend is consistent with results from a previous review by Abubakari et al. [10] in which West African women were 3 times more likely to be obese than their male counterparts. The higher prevalence of overweight and obesity among women than men in Ghana is also consistent with globally observed gender difference in overweight and obesity patterns [9].

Although, physiological pathways such as the differences in body fat distribution and the influence of gonadal steroids on appetite have sometimes been used to explain gender differences in anthropometric indices [100], the influence of behavioral and socio-cultural factors remain extremely important [101, 102]. In Ghana, women tend to settle for more sedentary occupations (e.g. table-top trading) and research has documented lower levels of physical activity among Ghanaian women than men. In a study of the predictors of overweight and obesity among a cohort of urban Ghanaian women, just around 21% were found to maintain adequate physical activity levels [54]. In a similar recent study among urban youth in Accra, about 4 in 5 (84.1%) persons were found to be physically inactive, with rates higher in females (94.7%) than males (70.5%) [103]. Additionally, studies have reported that many Ghanaian communities show great admiration towards large body size [59, 104]. Often, large body size is considered as a sign of “affluence” and women also tend to perceive this as constituting “beauty, good health and happiness in marriage” [54]. Anecdotal evidence suggest that in some Ghanaian communities with high HIV prevalence, the attainment of a large body size is sometimes misconstrued to mean a “virus-free” status and many individuals therefore desire large body size to avert any stigma. These socio-cultural construction of ideal body size may be implicated in the rising overweight and obesity burden in the country.

Our analysis also brings to bear the impacts of urbanization on the overweight/obesity epidemic in Ghana as confirmed by the near 1.6 and 2.6 fold increase in overweight and obesity prevalence, respectively among urban dwellers as compared to their rural counterparts. The differences in overweight/obesity prevalence in Ashanti, Northern, Central and Greater Accra regions also broadly mimic the extent of urbanization. Greater Accra, being the region that harbors the capital city has experienced the most rapid urbanization compared to any part of the country. In 2000 for instance, 87.4% of the population in Greater Accra region resided in urban centres compared to 53.2% for Ashanti and 27.0% for Northern regions [105]. Regardless, the extent of urbanization may not be the only underlying factor for the regional variation in overweight/obesity prevalence as cultural/tribal variations have been observed. Amoah [106], for instance reported highest overweight and obesity prevalence among the Akan and Ga tribes and relatively low rates among Ewes. Similar results were obtained by Agbeko et al. [107] after a secondary analysis of data from the 2008 DHS. The variations in overweight and obesity prevalence among the ethnic groups are thought to broadly reflect differences in social behaviors including work patterns, meal preparation and perception of body size.

Ghana like many other countries is also experiencing its fair share of the impacts of globalization. Over the last three (3) decades, the country has seen dramatic changes in telecommunication, transportation and exposure to the global market. Today, almost every household has someone owning a mobile phone compared to 1996 when there were just around three telephone lines per 1000 Ghanaians [14]. More Ghanaians now have access to internet and combined with other improved communication channels, exposure to foreign lifestyle and marketing has increased greatly. The effects of these is being witnessed in the form of drastic lifestyle changes including westernization of diet and high levels of sedentary lifestyle which have gained great momentum particularly in urban centres [14]. Studies have pointed out that “the nutrition transition and the rise in technology-aided, sedentary lifestyles (cars, computers at home and in internet cafes, games consoles for elite and middle class youth) are strongly implicated in Ghana’s obesity and chronic disease epidemics” [87]. Alcohol consumption have all been given significant boost and evidence suggests that Ghanaians who consume excessive alcohol have higher risks of overweight and obesity [60, 107]. Furthermore, as Ofori-Asenso and Garcia [14] discussed, the built environment in many urban centres in Ghana have not evolved to meet the expanding population; roads are usually congested and there are not many sidewalks or parks that could encourage physical activities like running and walking. These poor urban planning if not properly addressed could be a catalyst for further escalation of obesity in the country even as other factors drive an obesogenic environment. The prevalence of overweight/obesity in the rural areas reported in this review is also a call for concern and action as this is high compared to what has been reported in the past [32]. This highlights that the increasing obesity and overweight prevalence in Ghana may be more widely spread than previously thought. To fully explore the contribution of the urban environment to the obesity/overweight burden, further research could focus for instance on assessing how individuals’ risk profiles change once they migrate from rural to urban centres and vice versa.

While in many high-income countries, obesity tends to be more prevalent in persons with lower socioeconomic status (SES), the reverse has been the case in many low-income countries [108]. Studies by Amoah [106] and Appiah et al. [54] have documented higher prevalence of overweight/obesity in high-class Ghanaians compared with the low class residents. Additionally, Ghanaians with tertiary education have been found to have the highest prevalence of obesity compared with less literate and illiterate subjects. This may tie in to the perceptions of larger body size and affluence in many Ghanaian communities [59], but may also relate to the fact that increasing affluence may come with increased choice and accessibility to food and may promote intake of larger portion sizes [54, 109]. Moreover, evidence suggests that in Ghana, most fast-food joints and restaurants are crowded in wealthy neighborhoods and tend to target high-class clientele [110]. Agyei-mensah and de-Graft Aikins [87], also report that among the working (middle to high class) population in Ghana, “there is an emerging trend of individuals working late or hanging out at after-work bars to beat the heavy evening traffic; these practices are implicated in late eating and increased alcohol intake, and by extension increased chronic disease risks”.

The high and rising burden of overweight and obesity as documented in this study should be a concern to nutritional scientists, health workers and government of Ghana due to the impact on health and a possibility of a an explosion of chronic diseases such as hypertension, diabetes or even cancers [9]. Already, the prevalence of diseases like hypertension and diabetes are increasing significantly. Addo et al. [111], for instance, reported a high prevalence of hypertension in Ghana ranging from 19.3% in rural to 54.6% in urban areas with factors such as increased salt intake and increasing BMI being implicated in the rising prevalence. The prevalence of type-2 diabetes in Ghana in 2014 was estimated as 6% with over 450,000 persons living with the disease [14]. These rates are about 15 times high when compared to prevalence of about 0.4% in 1956 [112]. The impacts of overweight and obesity on individuals’ wellbeing and on productivity means that if the trends observed persists, the consequences on Ghana’s economic indicators can be enormous. Yet in Ghana, government’s effort continually focus on reducing hunger and in many instances neglect the growing problem of overweight/obesity. De graft Aikins [113], attributes this to longstanding misconception that NCDs and risk factors including obesity do not pose significant health challenges. To address the rising problem of overweight/obesity in Ghana, greater commitment from government will be needed to ensure efficient resource allocation and also to provide broader policy framework within which interventions can be implemented [114, 115]. Approaches adopted should be broad in nature and must not undermine undernutrition prevention efforts but should focus on tackling all aspects of malnutrition (over/undernutrition) to ensure a “sustainable nutrition for all” Ghanaians [116].

### Strengths and limitations

This review presents a stronger evidence regarding the prevalence of overweight/obesity among Ghanaian adults as it is based on larger number of studies than previously published reviews. Regardless, it has some limitations. Firstly, while the review covered studies that collected data from all regions of Ghana, there was a significant regional imbalance with 60% of studies conducted in two regions, Ashanti and Greater Accra alone. Subsequently, pooled estimates were not available for six (6) regions. The regional imbalance in data is likely to shift estimates as more evidence on overweight and obesity prevalence in the under-represented regions become available. Secondly, most studies did not report on the prevalence across broad socio-demographic characteristics such as religion, family size, marital status and ethnicity, although, these have all been identified as useful predictors of overweigh and obesity [117, 118]. Furthermore, a high level of heterogeneity across studies was observed including the presence of publication bias. The assessment of overweight/obesity among diabetes patients were also based on limited studies and dominated by those conducted in urban patients. Our temporal analysis of overweight/obesity prevalence was also based on studies’ publication years. A more robust approach for this analysis would have required the use of midpoint of actual study/sampling periods [119]. The use of publication period as opposed to actual period in which study/sampling was conducted may introduce some bias as there can be a time-lag between when a study is conducted is when it is published. In spite of the limitations outlined, the prevalence of overweight and obesity presented in this review should speak to current situation as most studies were recently published, including 79% of studies published within the last 5 years (2011–2016). The prevalence information on overweight and obesity presented should guide and inform policy makers in terms of resource allocation and planning towards controlling the rising burden of obesity and overweight in this country.

## Conclusions

Evidence available supports a high and rising prevalence of overweight and obesity among Ghanaian adults. This presents a significant public health issue, and the implications on current and future population health, burden of chronic diseases and on health care spending can be enormous for a country that is still battling many infectious and parasitic diseases. Further research is needed to provide greater insights into the major drivers underlying the rising overweight and obesity epidemic and also to offer context in terms of documenting the regional difference in prevalence. Urgent population-wide interventions and policy directions that assume broad approaches, are culturally acceptable, cost-effective and sustainable are clearly needed to tackle this emerging epidemic.

## Additional file

Additional file 1: PRISMA Checklist. (DOC 64 kb)

## Abbreviations

BMI: Body mass index
DHS: Demographic and Health Surveys
HDI: Human development index
LMICs: Low and middle income countries
MDG: Millennium Development Goals
NCDs: Non-communicable diseases
NHIS: National health insurance scheme
PRISMA: Preferred Reporting Items for Systematic Reviews and Meta-Analyses
SES: Socioeconomic status
WHO: World Health Organization
